# Photobiomodulation with a 660-Nanometer Light-Emitting Diode Promotes Cell Proliferation in Astrocyte Culture

**DOI:** 10.3390/cells10071664

**Published:** 2021-07-02

**Authors:** Sung-Ryeong Yoon, Namgue Hong, Min-Young Lee, Jin-Chul Ahn

**Affiliations:** 1 Department of Medical Laser, Graduate School of Medicine, Dankook University, Cheonan 31116, Korea; 72201437@dankook.ac.kr; 2 Medical Laser Research Center, College of Medicine, Dankook University, Cheonan 31116, Korea; hnk210@dankook.ac.kr; 3 Department of Otolaryngology-Head & Neck Surgery, College of Medicine, Dankook University, Cheonan 31116, Korea; 4 Beckman Laser Institute Korea, College of Medicine, Dankook University, Cheonan 31116, Korea

**Keywords:** astrocyte, proliferation, photobiomodulation, primary culture

## Abstract

Astrocytes act as neural stem cells (NSCs) that have the potential to self-renew and differentiate into other neuronal cells. The protein expression of these astrocytes depends on the stage of differentiation, showing sequential expression of multiple proteins such as octamer-binding transcription factor 4 (Oct4), nestin, glial fibrillary acidic protein (GFAP), and aldehyde dehydrogenase 1 family member L1 (aldh1L1). Photobiomodulation (PBM) affects cell apoptosis, proliferation, migration, and adhesion. We hypothesized that astrocyte proliferation and differentiation would be modulated by PBM. We used an optimized astrocyte culture method and a 660-nanometer light-emitting diode (LED) to enhance the biological actions of many kinds of cells. We determined that the 660-nanometer LED promoted the biological actions of cultured astrocytes by increasing the reactive oxygen species levels. The overall viability of the cultured cells, which included various cells other than astrocytes, did not change after LED exposure; however, astrocyte-specific proliferation was observed by the increased co-expression of GFAP and bromodeoxyuridine (BrdU)/Ki67. Furthermore, the 660-nanometer LED provides evidence of differentiation, as shown by the decreased Oct4 and GFAP co-expression and increased nestin and aldh1L1 expression. These results demonstrate that a 660-nanometer LED can modify astrocyte proliferation, which suggests the efficacy of the therapeutic application of LED in various pathological states of the central nervous system.

## 1. Introduction

The brain contains the following two kinds of cell: neurons and glial cells. Glial cells include astrocytes, microglia, and oligodendrocytes. Among glia cells, microglia belong to the monocyte/macrophage lineage, while astrocytes, belong to neural lineage. Astrocytes are the most abundant and have crucial roles in the brain.

Protein expression determines the characteristics of cells. Astrocytes differentiate from neural stem cells (NSCs) into mature cells, and protein expression starts with the embryonic rosette (octamer-binding transcription factor 4, Oct4) and goes through neural progenitors (nestin) to astrocytes (glial fibrillary acidic protein, GFAP) [1]. Oct4 is a neural progenitor transcription factor. Nestin is a cytoskeletal intermediate filament. It has been used as a marker for progenitor cells in the CNS. During maturation, nestin is gradually downregulated and replaced by tissue- or cell-specific intermediate filament proteins, such as GFAP and microtubule-associated protein 2 (MAP2) [2]. GFAP expression increases again in reactive astrocytes when an immune response occurs [3]. Therefore, GFAP is not an astrocyte marker, but has been used as a marker for immature and reactive astrocytes [4]. Expression of the astrocyte-specific proteins aldolase C (ALDOC), GLAST, aldh1L1, and GLT1 increases as astrocytes mature [5,6,7,8]. These proteins have been used as markers for mature astrocytes. Functionally, astrocytes support neuronal network activity and regulate synaptogenesis and neurotransmitters. They also maintain the blood–brain barrier and provide metabolic and trophic support, aiding neuron survival [9,10,11,12]

In neurodegenerative diseases, these astrocyte functions can be dysregulated [13], which can lead to excessive neuroinflammation and neuron death [14]. Therefore, studies of the relationships between astrocytes and neurodegenerative and brain diseases are important.

Photobiomodulation (PBM), a treatment method using infrared or near-infrared light (600–1100 nm), enhances cell proliferation, migration, and adhesion and promotes wound healing, tissue repair, chronic inflammation treatments, and hair regrowth [15]. PBM is being used in various fields related to neurodegenerative and brain diseases [16]. The latest study reported that PBM leads to a reduction in Aβ proportion via activation of the PKA/SIRT1 signaling pathway in Alzheimer’s disease transgenic mouse. It results in the reversal of spatial learning and memory impairments [17]. Other studies have shown that PBM rescues dendritic atrophy in Alzheimer’s disease by upregulating brain-derived neurotrophic factor (BDNF) [18]. In the case of 660-nanometer PBM, it reduces oxidative stress and induces BDNF expression in the hippocampus [19]. Furthermore, PBM regulates microglial function in neurodegenerative diseases [20]. Additionally, PBM rescues stress-induced neuroglial damage and glutamate uptake improvement by upregulating the GLT-1 of astrocyte [21]. While many studies of PBM treatment have focused on neurons, few have examined the effects of PBM on astrocytes.

In this study, we assessed whether a 660-nanometer light-emitting diode (LED) promoted astrocyte proliferation and differentiation and examined the effects of PBM on regulating neurotrophic factors in cortical astrocytes.

## 2. Materials and Methods

### 2.1. Ethics Approval

All animal experiments were conducted according to the guidelines of the Declaration of Helsinki and approved by the Institutional Animal Care and Use Committee (IACUC; No. DKU-21-023; 12 May 2021) of Dankook University, Republic of Korea. All experiments were performed in accordance with relevant guidelines and regulations. This study is reported in accordance with the ARRIVE guidelines. All animals were euthanized with urethane dissolved in tertiary distilled water (12.5%, 0.8 mL/100 g)

### 2.2. LED Irradiation (PBM)

**Figure 1:**
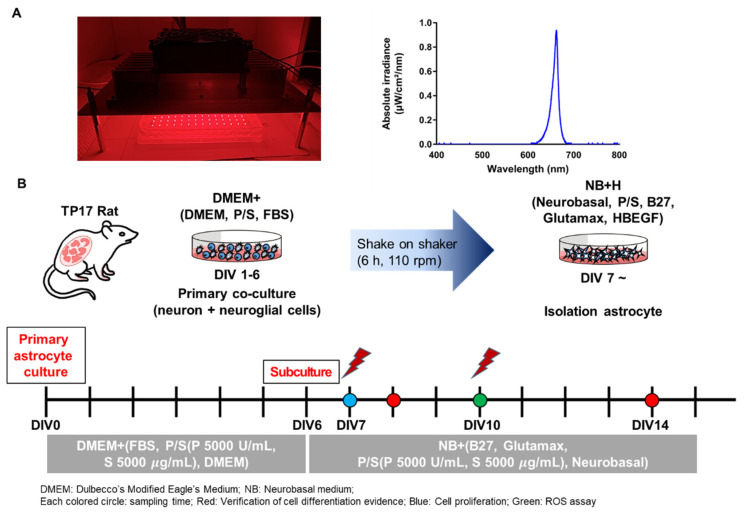
The 660-nanometer LED Irradiation Schedule. (**A**) Representative image of LED irradiation. Graph showing that the LED irradiation was delivered at 660 nm; (**B**) Schema of the astrocyte culturing method and experimental schedule. From DIV0 to DIV6, cells were incubated in DMEM+ (DMEM, FBS, P/S [P = 5000 U/mL, S = 5000 μg/mL]). On DIV6, cells were subcultured for isolation of astrocytes. Cells were incubated in NB+ medium (Neurobasal, B27, GlutaMAX, P/S (P = 5000 U/mL, S = 5000 μg/mL)). The LED irradiation protocol was divided into the following two methods: irradiation on DIV7 only, and irradiation on both DIV7 and DIV10. Each colored circle denotes the sampling time for several experiments. The red circle indicates verification for cell differentiation evidence, and the blue and green circles indicate cell proliferation and ROS assay, respectively.

To study the effects of LED on astrocytes, a 660-nanometer LED was used (Figure 1A) with a power of 10 mW/cm^2^, duration of 10 min, and energy density of 6 J/cm^2^ (Table 1). Energy density was determined by referring to previous studies dealing with 660-nanometer PBM [22,23,24]. Cells were irradiated with the 660-nanometer LED in the dark. The LED irradiation schedule was fixed on day in vitro (DIV) 7 and DIV10, which are developmental stages of cultured astrocytes (Figure 1B). The 660-nanometer LED was exposed twice to expose the maximum energy within the given conditions. To measure the light source power density, a power meter (PD300-TP-ROHS, Ophir PD, Jerusalem, Israel) was used.

### 2.3. Cell Culture

Cultured astrocytes were prepared as reported previously [25] with minor modifications. Briefly, brains were extracted from Sprague Dawley rats at embryonic day 17. Cortices were separated from extracted brain tissues and incubated in a water bath at 37 °C with 0.25% trypsin for 20 min, followed by gentle trituration.

Dissociated cells were counted and plated on 0.04% polyethylenimine (PEI)-coated culture dishes (4,000,000 cells/60 mm dish) with culture media (10% fetal bovine serum, 5000 U/mL penicillin, and 5000 μg/mL streptomycin in Dulbecco’s modified Eagle’s medium; Gibco, Thermo Fisher Scientific, Waltham, MA, USA). Six days after the culture, the dishes were shaken at 110 rpm for 6 h. After shaking, cells were treated with 0.25% trypsin and plated on a 0.04% PEI-coated microplate or 18-mm coverslip in a 12-well plate (20,000 cells/plate), with heparin-binding epidermal growth factor (HB-EGF) (50 μg/mL) in Neurobasal Medium (Gibco) containing 2% B27 supplement (Gibco), 2 mM GlutaMAX (Gibco), 5000 U/mL penicillin, and 5000 μg/mL streptomycin (Gibco).

### 2.4. Cell Viability Assay

A Cell Counting Kit-8 (CCK-8; Dojindo Laboratories, Kumamoto, Japan) was used for analyzing cell viability. Cells were prepared in a 96-well microplate at a density of 1 × 10^4^ cells/well. A CCK-8 reagent was added to cells 24 h after 660-nanometer LED irradiation and incubated for 2 h at 37 °C. After 2 h, the absorbance of cells was measured at 450 nm with a microplate reader. Cell viability (%) was calculated by determining the optical density of LED-irradiated cells compared to control cells.

### 2.5. ROS Assay

For the reactive oxygen species (ROS) assay, the DCFDA/H2DCFDA Cellular ROS Assay Kit (ab113851; Abcam, Cambridge, UK) was used. Cells were plated on an 18-mm coverslip in a 12-well plate at a density of 2 × 10^4^ cells/well and irradiated with the 660-nanometer LED on DIV7 and DIV10. On the last day of irradiation, the medium was removed immediately after 660-nanometer LED irradiation and the cells were washed with 1× supplemented buffer (equilibrated at 37 °C before use) at least twice. Then, 20 mM DCFDA solution was diluted in 1× supplemented buffer to a final concentration of 20 μM. Diluted DCFDA solution was added to stain the cells. Cells were incubated for 45 min at 37 °C in the dark and then washed with 1× buffer once or twice. Live cell fluorescent microscopy was performed. Oxidation derivative of DCFDA (2′,7′—dichlorofluorescein, DCF) is fluorescent and it is detected by ex/em at 485 nm/535 nm, as it is FITC. To avoid photobleaching, low-light or dark conditions were maintained.

### 2.6. Immunocytochemistry

Cells were fixed in methyl alcohol and permeabilized with 0.3% Triton X-100 in phosphate-buffered saline (PBS). After blocking in 10% bovine serum albumin (BSA), cells were incubated overnight at 4 °C with the following primary antibodies: anti-GFAP (MAB360; EMD Millipore, Burlington, MA, USA), anti-aldh1L1 (NBP2-50045; Novus Biologicals, Littleton, CO, USA), anti-nestin (ab92391; Abcam), anti-Oct4 (ab18976; Abcam), anti-neuronal nuclear protein (NeuN) (MAB377; EMD Millipore), anti-MAP2 (M9942; Sigma-Aldrich, St. Louis, MO, USA and ab32454; Abcam), anti-ionized calcium-binding adaptor molecule 1 (Iba1) (PA5-27436; Invitrogen, Carlsbad, CA, USA and NB100-1028; Novus Biologicals), anti-bromodeoxyuridine (BrdU) (B5002; Abcam), anti-Ki67 (ab15580; Abcam), anti-BDNF (ab108319; Abcam), and anti-nerve growth factor (NGF) (ab52918; Abcam). After treatment with primary antibodies, the cells were washed with PBS and then incubated with secondary antibodies conjugated with Alexa488, Alexa555 (A11001, A22428; Invitrogen), and Alexa647 (ab150135; Abcam) for 1.5 h at room temperature. After washing with PBS, immunostained cells were mounted with VECTASHIELD^®^ Antifade mounting medium with DAPI (H-1200; Vector Laboratories, Burlingame, CA, USA) and observed with a confocal microscope (FV31-S; Olympus, Tokyo, Japan).

### 2.7. Western Blot Analysis

Cells in a 6-well plate (1 × 10^6^ cells/well) were irradiated with a 660-nanometer red LED. Proteins were extracted in an RIPA buffer (R2002; Biosesang, Seoul, Korea) supplemented with protease and phosphatase inhibitors. After centrifugation (4 °C, 13,000 rpm, 15 min), the supernatant was collected into a new 1.5-milliliter tube and stored at −80 °C. Proteins were resolved on SDS-PAGE gels, and electrotransferred to polyvinylidene fluoride membranes. Then, the membranes were blocked in TBST (10 mM Tris- HCl, pH 7.4, 150 mM NaCl, 0.1% Tween 20) containing 5% BSA and incubated with the following appropriate primary antibodies: anti-TNFα (ab199013; Abcam), anti-IL-6 (ab208113; Abcam), anti-IL-1β (ab205924; Abcam), anti-Oct4 (ab18976; Abcam), anti-nestin (ab92391; Abcam), anti-aldh1L1 (NBP2-50045; Novus Biologicals), anti-BDNF (ab108319; Abcam), and anti-nerve growth factor (NGF) (ab52918; Abcam) and secondary antibodies. β-Actin (A1978; Sigma-Aldrich) and a β-tubulin antibody (86298S; Cell signaling Technologies, Danvers, MA, USA) were used as the standard. Signals were detected with the Clarity™ Western ECL Substrate (170-5060; Bio-Rad, Hercules, CA, USA). Bands were captured using Image Lab 6.0.1 (Bio-Rad). The Western blots were quantified using ImageJ software (version 1.53a, National Institutes of Health [NIH], Bethesda, MD, USA). Densitometry analyses are presented as the ratio of protein to β-actin or β-tubulin protein, which was compared to that of the controls and normalized to 1.

### 2.8. Statistical Analysis

Images were analyzed with ImageJ software (NIH). To analyze the expression pattern of proteins related to NSCs, astrocytes, and cell proliferation, cells were manually counted in the region of interest. Statistical analyses were performed using Prism v7.0 (GraphPad, La Jolla, CA, USA). All experimental and control data are presented as means ± standard errors of the mean. The Shapiro–Wilk normality test was used to determine whether the data followed a Gaussian distribution. When it did, two-tailed unpaired *t*-tests were performed; otherwise, the Mann–Whitney U test was used.

## 3. Results

### 3.1. Verification of the Astrocyte Culture

**Figure 2:**
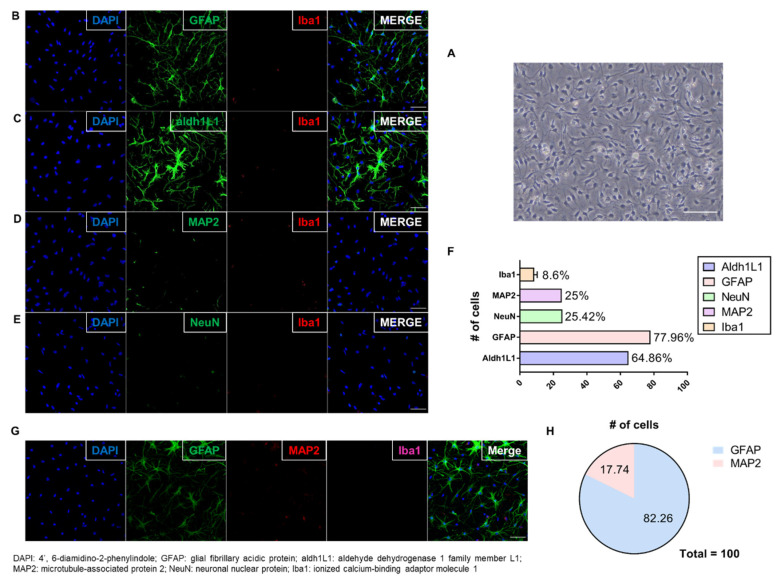
Verifying the Culture of Astrocytes. (**A**) Differential Interference Contrast microscopy image showing cultured astrocytes with stellate morphology on DIV14 (scale bar: 50 μm); (**B**–**E**) Dual immunofluorescent image showing that most cells expressed astrocyte markers (GFAP and aldh1L1); fewer expressed Iba1 (microglia), NeuN, and MAP2 (neurons) (scale bar: 50 μm); (**F**) Among every 100 cells, 60–80 expressed astrocyte markers (aldh1L1 or GFAP), and 20 expressed neuronal cell markers (NeuN or MAP2). The remaining cells expressed a microglia marker (Iba1). (No error bar due to one cell type group each); (**G**) Triple immunofluorescent image showing that most cells expressed GFAP (astrocytes); fewer expressed MAP2 (neurons). Expression of Iba1 (microglia) was not detected (scale bar: 50 μm); (**H**) Plots analysis showing similar results to dual staining. Microglia (Iba1) was not plotted because of no detection. The graph shows that the cell culture in this study was dominated by astrocytes.

A protocol was used for culturing astrocytes that establishes in vivo morphology and gene expression [25,26], and allows astrocytes to be cultured in a two-dimensional in vivo environment [26]. The astrocytes were cultured according to a previous study with minor modifications (Figure 1B) [25]; the cultured astrocytes are shown in Figure 2A. To verify the culture of astrocytes, they were immunostained with markers for astrocytes (GFAP and aldh1L1), neurons (NeuN and MAP2), and microglia (Iba1). Dual immunostained images showed that most of the cells expressed the astrocyte markers GFAP and aldh1L1. Cultured astrocytes have a star shape, which is considered in vivo-like morphology. A few cells expressed other markers, such as NeuN, MAP2, and Iba1 (Figure 2B–E). In total, 60–80 of every 100 cells expressed astrocyte markers (aldh1L1 or GFAP), and 20 expressed neuronal cell markers (NeuN or MAP2). The remaining cells expressed a microglia marker (Iba1) (Figure 2F). In the case of microglia, it accounts for 8.6%, and this amount can release large quantities of agents and affect the astrocytes. Furthermore, there are previous studies in which pro-inflammatory cytokines’ (e.g., TNFα, IL-6, IL-1β) release of microglia is decreased by PBM [27,28]. Therefore, to confirm that microglia had no effect on this experiment, the level of cytokines was measured through Western blot (Supplementary Figures S1 and S2). As a result, there were no significance differences, confirming that the effect of inflammatory response by microglia is negligible. In addition, in the triple staining, astrocytes (GFAP) and neurons (MAP2) showed similar results to dual staining, but microglia (Iba1) were not detected (Figure 2G,H). These results showed that astrocytes were cultured successfully.

### 3.2. Astrocyte Proliferation was Exclusively Enhanced by 660-nm LED Irradiation

**Figure 3:**
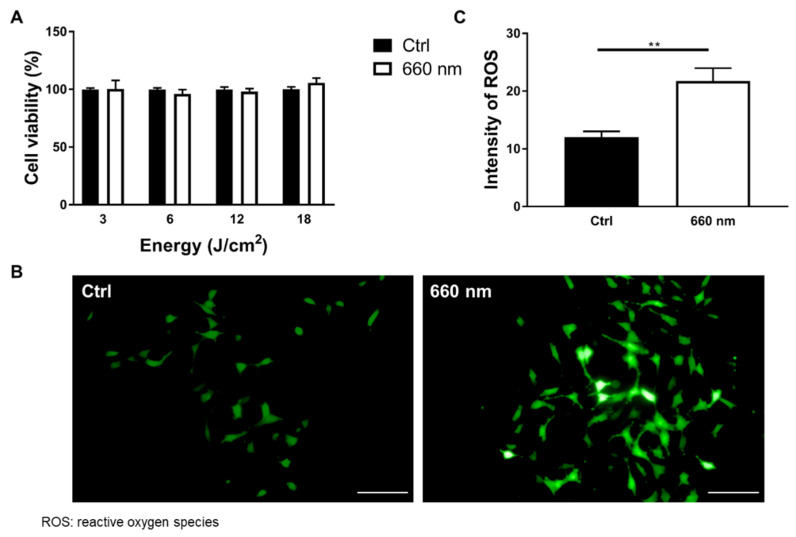
Alteration of Cell Viability and ROS Expression Induced by 660-nanometer LED Irradiation. (**A**) Cell viability (%) analyses after 24 h of 660-nanometer LED irradiation with a power of 10 mW/cm^2^ and various total energies (3, 6, 12 and 18 J/cm^2^). Results showed that there were no significant effects on overall cell types at any power density (*p* > 0.05; *n* = 9); (**B**) Representative images of the ROS assay (FITC) (scale bar: 100 μm); (**C**) The intensity of ROS-positive (FITC+) cells was significantly increased by LED irradiation (Ctrl vs. 660 nm; *n* = 48 vs. 57, ** *p* < 0.01).

PBM at 660 nm regulates the biological actions of many types of cells, including diabetic wounded cells, fibroblasts, and human adipose-derived stem cells [24,29,30,31]. In the astrocyte culture, DIV7–DIV14 is considered the developmental stage, and astrocytes before DIV14 still have neural stem cell (NSC) features [26]. Therefore, irradiation was conducted on DIV7 and DIV10 to determine whether the 660-nanometer LED affected the developmental stage of the astrocyte culture (Figure 1B). Two exposures were the most appropriate conditions for a period between DIV7 and DIV14 in order to expose the maximum amount of light at regular intervals. To determine whether the 660-nanometer LED causes toxicity or an increase in overall cell proliferation, a cell viability assay was performed in an astrocyte culture, which includes various cell types other than astrocytes (*n* = nine wells). The cells were irradiated with a total density ranging from 3 to 18 J/cm^2^. The results showed that the 660-nanometer LED had no effects on the overall cell density at any power density (Figure 3A). Once the cells were stimulated by PBM, cytochrome c oxidase (CCO) in the mitochondria respiratory chain was activated, leading to a membrane potential (MMP) increase. It leads to dissociation of inhibitory nitric oxide from CCO. As a result, enzyme activity, ATP production, and reactive oxygen species (ROS) were increased [32,33]. In this study, to evaluate whether LED affects cellular activity, an ROS assay, in which ROS expression was altered by LED irradiation [34], was performed. The cells were irradiated by the 660-nanometer LED on DIV7 and DIV10.

**Figure 4:**
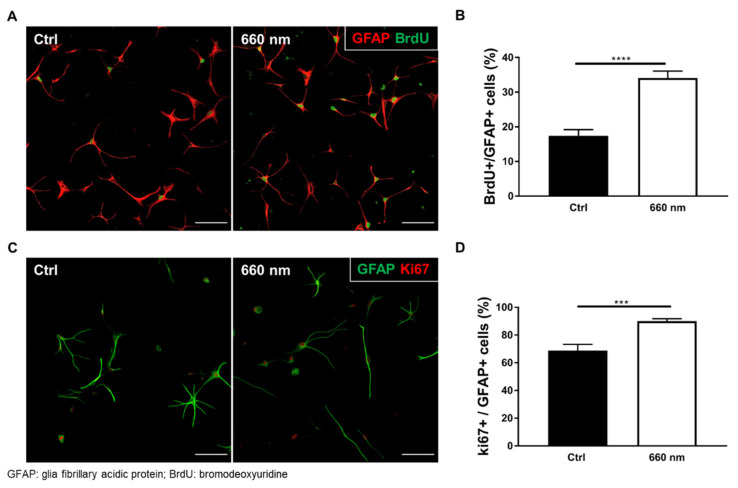
Astrocyte Proliferation was Promoted by 660-nanometer LED Irradiation. (**A**) Confocal image showing immunofluorescent staining (red, GFAP (astrocytes); green, BrdU (DNA duplication)) (scale bar: 100 μm); (**B**) The percentage of BrdU- and GFAP-positive cells was significantly increased by 660-nanometer LED irradiation (Ctrl vs. 660 nm; *n* = 58 vs. 55, **** *p* < 0.0001); (**C**) Confocal image showing immunofluorescent staining (red, Ki67 (proliferation); green, GFAP (astrocytes)) (scale bar: 100 μm); (**D**) The percentage of Ki67- and GFAP-positive cells was significantly increased by LED irradiation (*n* = 45, *** *p* < 0.001).

On DIV10, 20 μM DCFDA solution was treated immediately after irradiation and incubated for 45 min at 37 °C in the dark. ROS expression was significantly increased by 660-nanometer LED irradiation (Mann–Whitney U test; control vs. 660 nm; *n* = 48 vs. 57; ** *p* < 0. 01; *p* = 0.0083; U = 960; Figure 3B,C). To evaluate cell-specific proliferation, BrdU staining was performed after 660-nanometer LED irradiation, as BrdU labels proliferating cells. Cells were fixed after 2 h of BrdU treatment. The percentage of BrdU-positive astrocytes (BrdU^+^/GFAP^+^) was significantly increased after 660-nanometer LED irradiation (Mann–Whitney U test; control vs. 660 nm; *n* = 58 vs. 55; **** *p* < 0.0001; U = 635; Figure 4A,B). To confirm this cell-specific proliferation, Ki67 staining was also performed. The percentage of Ki67-positive astrocytes (Ki67^+^/GFAP^+^) was significantly increased (Mann–Whitney U test; *n* = 45; *** *p* < 0.001; *p* = 0.0005; U = 597; Figure 4C,D). These results suggest that 660-nanometer LED irradiation specifically increases astrocyte proliferation.

### 3.3. Possibility of Astrocyte Differentiation by 660-nm LED Irradiation

**Figure 5:**
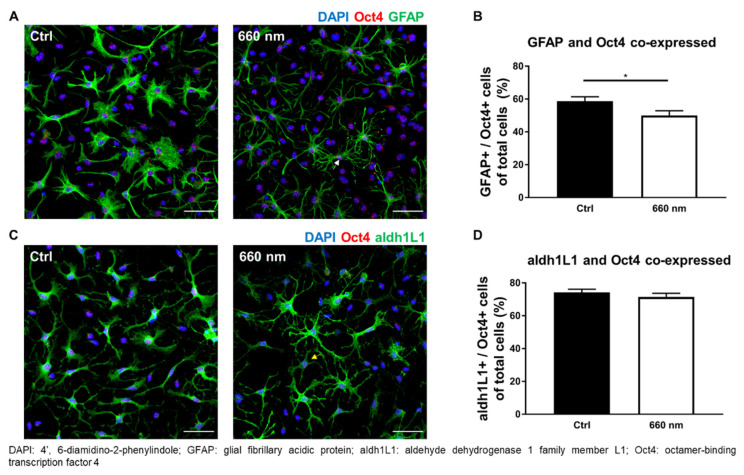
Astrocytic Differentiation Could be altered by 660-nanometer LED Irradiation (Oct4 co-expression). (**A**,**C**) Representative images of immunofluorescence staining (scale bar: 50 μm); (**A**) The white arrow denotes GFAP− and Oct4 co-expressing cells, which were significantly decreased by 660-nanometer LED irradiation; (**C**) The yellow arrow denotes aldh1L1− and Oct4 co-expressing cells; (**B**,**D**) The graph of protein expression was shown. Compared to the control, 660-nanometer LED irradiation significantly decreased GFAP+/Oct4+ cell expression (*n* = 35, * *p* < 0.05) and aldh1L1+/Oct4 showed only a decreased tendency.

The enhancement of cell proliferation by the 660-nanometer LED was confirmed. Not only cell proliferation but also cell differentiation, especially stem cell differentiation, can be regulated by PBM [35,36,37,38,39,40]. The protein expression pattern of cells differs according to the differentiation stage. In the case of astrocytes, GFAP, nestin, and Pax6, among other markers, are expressed in progenitor cells. In mature astrocytes, aldh1L1, aldolase, fructose-bisphosphate C, cluster of differentiation 44, and excitatory amino acid transporter 1 are expressed [1]. Cells were immunostained with pluripotent and NSC markers (Oct4 and nestin, respectively), developmental marker (GFAP), and maturity marker (aldh1L1) after twice being irradiated with the 660-nanometer LED, to determine whether the LED regulated the differentiation of astrocytes. The co-expression of Oct4 or nestin with GFAP or aldh1L1 (markers of immature or mature astrocytes; Table 2) were analyzed. The expression of cells co-expressing GFAP (developing astrocytes) and Oct4 (pluripotent stem cells) was significantly decreased by the 660-nanometer LED irradiation (Mann–Whitney U test; *n* = 35; * *p* < 0.05; *p* = 0.0240; U = 421; Figure 5A,B) (Table 2).

**Figure 6:**
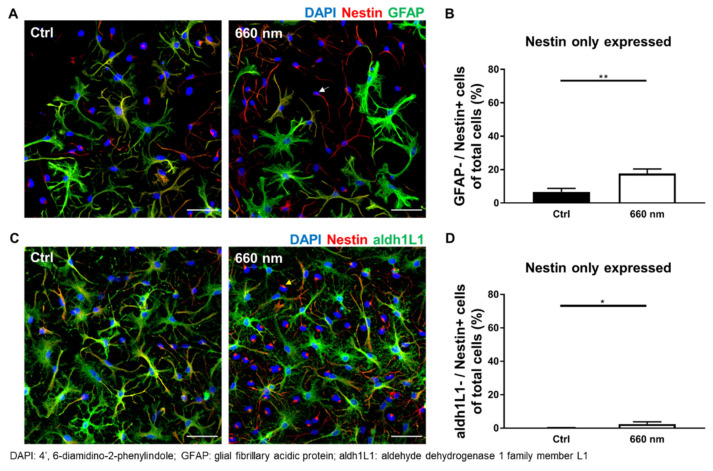
Astrocyte Differentiation Could be Altered by 660-nanometer LED Irradiation (nestin co-expression). (**A**,**C**) Representative images of immunofluorescence staining. The white and yellow arrow denotes nestin-expressing cells, which were significantly increased by 660-nanometer LED irradiation (scale bar: 50 μm); (**B**,**D**) Only the graph of significant protein expression was shown. GFAP−/Nestin+ and aldh1L1−/Nestin+ expression levels were significantly increased compared to the control (*n* = 38 and 39, * *p* < 0.05, ** *p* < 0.01).

The number of cells co-expressing aldh1L1 (mature astrocytes) and Oct4 (pluripotent stem cells) was non-significantly decreased (*p* > 0.05; Figure 5C,D; Table 2). Another NSC marker with more widespread expression during differentiation, nestin, was used to confirm possibility of differentiation by 660-nanometer LED irradiation; only nestin-positive cells were significantly increased by irradiation (Mann–Whitney U test; (GFAP^−^/Nestin^+^) and (aldh1L1^−^/Nestin^+^); *n* = 38 and 39; * *p* < 0.05, ** *p* < 0.01; *p* = 0.0057 and 0.0335; U = 462.5 and 640.5; Figure 6A–D). The expression of cells positive for both nestin and GFAP was decreased, but not significantly (*p* > 0.05; Table 2). In the case of mature astrocytes, aldh1L1-positive cell expression tended to increase, but not significantly (*p* > 0.05; Table 2). Overall, these results show the decreased co-expression of Oct4 and increased expression of nestin, suggesting the possibility of differentiation. Western blot analysis was also performed to confirm the final differentiation possibility. The results showed that 660-nanometer LED irradiation did not change Oct4 expression, while the expression of aldh1L1 and nestin was significantly increased (two-tailed unpaired *t*-test and Mann–Whitney U test; *n* = 4; ** *p* < 0.01 and * *p* < 0.05; *p* = 0.0087 and 0.0286; t = 3.822; df = 6 and U = 0, respectively; Supplementary Figures S3 and S4). More cells differentiated into mature astrocytes after 660-nanometer LED irradiation compared to the control group (Figure 5 and Figure 6), suggesting that 660-nanometer LED irradiation can provide evidence for cell differentiation. In addition, an abundance of astrocyte processes was seen post-irradiation (Figure 5 and Figure 6). Astrocyte processes wrap around individual synapses, and an increased number of processes better supports neuronal cell structures via synaptogenesis and neurotransmitter recycling [41,42,43,44]. However, there is possibility that these results are induced by the proliferation of specific cell types not by differentiation.

### 3.4. Alteration of Neurotrophic Factors in Astrocyte Culture by 660-nm LED Irradiation

LED irradiation can increase the release of growth factors, which in turn promotes cell proliferation and differentiation [45]. Several neurotrophic factors are expressed in the brain, including NGF, BDNF, and neurotrophin-3/-4. Neurotrophic factors promote cell survival and differentiation [46]. Therefore, we hypothesized that the regulation of cell proliferation by 660-nanometer LED irradiation might occur through the release of neurotrophic factors. To identify key neurotrophic factors for cell proliferation and differentiation, cells were fixed for 2 h after irradiation, where 2 h is the optimal reaction time of the neurotrophic factors tested.

**Figure 7:**
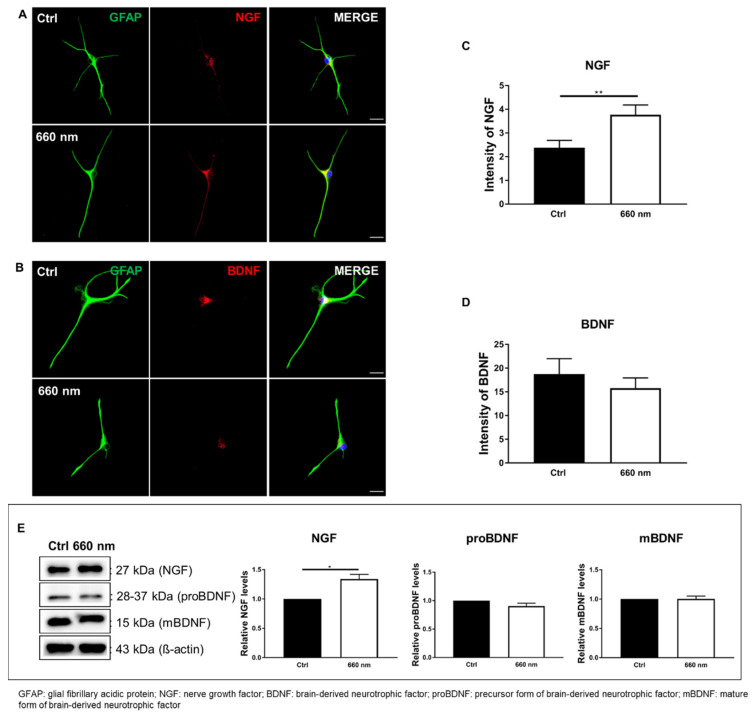
Alteration of NGF by 660-nanometer LED Irradiation. (**A**,**B**) Representative GFAP (green), NGF, and BDNF (red) immunostaining images (Scale bar: 25 μm); (**C**,**D**) NGF and BDNF analyses; (**C**) The intensity of NGF was significantly increased by 660-nanometer LED irradiation compared to control cells (*n* = 30, ** *p* < 0.01); (**D**) BDNF expression was decreased by 660-nanometer LED irradiation, but not significantly; (**E**) Western blot analysis of neurotrophic factors. In accordance with the immunofluorescence data, the expression levels of neurotrophic factors were altered by 660-nanometer LED irradiation, especially NGF, which was significantly increased (*n* = 4, * *p* < 0.05). Full-length blots/gels are presented in Supplementary Figure S5.

Immunostaining of representative neurotrophic factor markers, NGF and BDNF, was then performed. To confirm the increase in neurotrophic factors, the marker intensity was analyzed. The results showed that the intensity of NGF was significantly increased by 660-nanometer LED irradiation (Mann–Whitney U test; *n* = 30; ** *p* < 0.01; *p* = 0.0053; U = 263.5; Figure 7A,C), while the intensity of BDNF was non-significantly decreased (Mann–Whitney U test; *n* = 30; *p* > 0.05; *p* = 0.7412; U = 427; Figure 7B,D). To confirm the immunostaining results, Western blot analysis was performed. No difference in the protein expression of the precursor pro-BDNF and mature BDNF was observed between the control cells and the 660-nanometer LED-irradiated cells. However, the protein level of NGF was significantly increased by LED irradiation (Mann–Whitney U test; *n* = 4; * *p* < 0.05; *p* = 0.0286; U = 0; Figure 7E, Supplementary Figure S5). These results indicate that the LED-mediated increase in cell proliferation was regulated by the increased NGF level, suggesting that upregulation of the biological activity of astrocytes after 660-nanometer LED irradiation might occur through NGF.

## 4. Discussion

This study showed that ROS expression was increased by 660-nanometer LED irradiation. Immunostaining showed that cell proliferation and the possibility of differentiation were also increased by irradiation, which was confirmed using Western blot analyses. To determine the underlying mechanisms, we focused on the representative neurotrophic factors (BDNF and NGF) that can be increased by LED irradiation [45,47]. To this end, Western blotting and immunostaining were performed, and the results showed that the NGF protein expression was significantly increased by 660-nanometer LED irradiation; this suggests that the irradiation affected cell proliferation and differentiation possibility by upregulating the neurotrophic factor, NGF. Alternatively, PBM promotes DNA and RNA synthesis, and also increases protein production. Moreover, it plays a role in regulating the enzymatic activity and the pH inside and outside cells, and promotes cellular metabolism [48,49]. These effects of PBM might be related to the proliferation and differentiation possibility of astrocytes by PBM.

This study confirmed that PBM increased astrocyte proliferation, while the viability of all cells did not differ between the control and 660-nanometer groups. Contrary to some studies reporting that PBM increases cell viability, we found no difference in cell viability. This might have been because our culture system was dominated by astrocytes; therefore, the other cells might not have been healthy, and most neuron cell-related research has focused on pathological situations and mainly used 800-nanometer LEDs [50,51].

Cell proliferation is driven by cytochrome c oxidase (CCO), the first photoreceptor of red and near-infrared light. However, a previous study showed that near-infrared, not red, light predominantly affected mitochondrial cytochrome oxidase [52]. Another recent study suggested that 660-nanometer PBM does not require CCO to enhance cell proliferation [53]. In our study, a 660-nanometer LED significantly increased both astrocyte-specific proliferation and the ROS level. Therefore, we speculate that cell proliferation was increased via ROS, suggesting that ROS production is involved in the mechanism related to the effects of a red LED on cell proliferation. More research is needed to answer these questions.

In addition to cell proliferation and migration, the expression of various genes associated with cytokines and growth factors is induced by PBM [54,55]. We hypothesized that the growth factors involved in cell survival and differentiation mediate the proliferation and differentiation induced by PBM [46,56]. In this study, the expression of growth factors was altered by PBM; in particular, NGF protein expression was significantly increased by 660-nanometer LED irradiation, consistent with a previous study showing that PBM increases cell proliferation and NGF gene expression in human Schwann cells [57]. In addition, a previous study showing that NGF increases the number of cultured astrocytes [58] also posited that PBM-induced increases in NGF expression affect astrocyte proliferation. However, since the receptors and roles of NGF are diverse, more studies are needed regarding the specific mechanism underlying PBM-induced astrocyte expression.

Furthermore, we examined changes in astrocytes induced by PBM. The astrocyte culture contained HB-EGF to induce astrocytes in vivo and aid cell growth. Previous studies have suggested that HB-EGF can cause de-differentiation in the cell culture [25,26]. Therefore, it was unclear whether the evidence of cell differentiation in this study was related to HB-EGF. Further studies need to develop an improved culture technique without HB-EGF.

In conclusion, this study demonstrated that 660-nanometer LED irradiation affects the proliferation of cultured astrocytes and provides evidence of differentiation. The results suggested that astrocyte characteristics can be affected by 660-nanometer LED irradiation. Based on our findings, PBM can be expected to be a useful treatment for brain disorders and brain cell damage due to its interaction with astrocytes. In addition, this study provides further insight into the effects of PBM on astrocytes.

## Figures and Tables

**Table 1:** The parameters of light emitting diodes (LEDs).

Parameter	Value
Wavelength (nm)	660
Operating Mode	Continuous wave
Voltage Value (V)	25.2
Distance of LED from Cell Culture Plate (cm)	4
Irradiance (SUM) (mW/cm^2^)	10
Exposure Duration (min)	10
Radiant Energy (J/cm^2^)	6

**Table 2:** Demonstration of Presented Cell Types Depends on Protein Expression. Glial fibrillary acidic protein: GFAP; aldehyde dehydrogenase 1 family member L1: aldh1L1; octamer-binding transcription factor 4: Oct4U: Mann–Whitney U-test; t: Unpaired *t*-test; (* *p* < 0.05; ** *p* < 0.01).

Cell Type	Protein Expression	Ctrl vs. 660 nm		
		Mean ± SEM (# of Cells)	*p*-Value	U or t
Pluripotent stem cell	Oct4+/GFAP−	16.36 ± 3.026 vs. 22.67 ± 2.826 (35)	0.0595	452
	Oct4+/aldh1L1−	2.166 ± 0.6865 vs. 2.735 ± 0.6442 (36)	0.1542	531.5
Neural stem cell	Nestin+/GFAP−	6.725 ± 2.072 vs. 17.7 ± 2.648 (38)	0.0057 **	462.5
	Nestin+/aldh1L1−	0.1517 ± 0.1425 vs. 2.446 ± 1.3 (39)	0.0335 *	640.5
Developmental astrocytes	GFAP+/Oct4−	2.5 ± 0.6376 vs. 3.343 ± 0.5668 (35)	0.1681	495
	GFAP+/Nestin−	1.782 ± 0.2909 vs. 1.497 ± 0.2142 (39)	0.8988	709.5
Maturity astrocytes	aldh1L1+/Oct4−	8.484 ± 1.474 vs. 9.874 ± 1.694 (36)	0.1609	523
	aldh1L1+/Nestin−	5.219 ± 0.9469 vs. 9.785 ± 1.786 (39)	0.0617	573.5
Immature astrocytes with ability to differentiate to other specific cell types	GFAP+/Oct4+	58.78 ± 2.59 vs. 49.98 ± 2.88 (35)	0.0240 *	421
	GFAP+/Nestin+	56.76 ± 2.474 vs. 52.57 ± 3.059 (38)	0.2901	1.065
Mature astrocytes with ability to differentiate to other specific cell types	aldh1L1+/Oct4+	74.23 ± 1.921 vs. 71.37 ± 2.345 (36)	0.5177	590
	aldh1L1+/Nestin+	70.99 ± 1.686 vs. 66.17 ± 2.435 (39)	0.1907	629

## Data Availability

Not applicable.

## Supplementary Materials

The following are available online at https://www.mdpi.com/article/10.3390/cells10071664/s1, Figure S1: Western blot analysis of pro-inflammatory cytokines (*n* = 4); Figure S2: Full-length blots of pro-inflammatory cytokines; Figure S3: Western blot analysis of proteins related to differentiation of astrocyte (*n* = 4; * *p* < 0.05; ** *p* < 0.01); Figure S4: Full-length blots of differentiation of astrocyte Western blot analysis; Figure S5: Full-length blots of neurotrophic factor proteins Western blot analysis.

## References

[1] Engel M., Do-Ha D., Muñoz S.S., Ooi L. (2016). Common pitfalls of stem cell differentiation: A guide to improving protocols for neurodegenerative disease models and research. Cell. Mol. Life Sci..

[2] Bernal A., Arranz L. (2018). Nestin-expressing progenitor cells: Function, identity and therapeutic implications. Cell. Mol. Life Sci..

[3] Brenner M., Messing A. (2021). Regulation of GFAP expression. ASN Neuro.

[4] Li J., Khankan R.R., Caneda C., Godoy M.I., Haney M.S., Krawczyk M.C., Bassik M.C., Sloan S.A., Zhang Y. (2019). Astrocyte-to-astrocyte contact and a positive feedback loop of growth factor signaling regulate astrocyte maturation. Glia.

[5] Kugler P., Schleyer V. (2004). Developmental expression of glutamate transporters and glutamate dehydrogenase in astrocytes of the postnatal rat hippocampus. Hippocampus.

[6] Regan M.R., Huang Y.H., Kim Y.S., Dykes-Hoberg M.I., Jin L., Watkins A.M., Bergles D.E., Rothstein J.D. (2007). Variations in promoter activity reveal a differential expression and physiology of glutamate transporters by glia in the developing and mature CNS. J. Neurosci..

[7] Cahoy J.D., Emery B., Kaushal A., Foo L.C., Zamanian J.L., Christopherson K.S., Xing Y., Lubischer J.L., Krieg P.A., Krupenko S.A. (2008). A transcriptome database for astrocytes, neurons, and oligodendrocytes: A new resource for understanding brain development and function. J. Neurosci..

[8] Yang Y., Vidensky S., Jin L., Jie C., Lorenzini I., Frankl M., Rothstein J.D. (2011). Molecular comparison of GLT1+ and ALDH1L1+ astrocytes in vivo in astroglial reporter mice. Glia.

[9] Sofroniew M.V., Vinters H.V. (2010). Astrocytes: Biology and pathology. Acta Neuropathol..

[10] Verkhratsky A., Nedergaard M. (2018). Physiology of Astroglia. Physiol. Rev..

[11] Khakh B.S., Sofroniew M.V. (2015). Diversity of astrocyte functions and phenotypes in neural circuits. Nat. Neurosci..

[12] Santello M., Toni N., Volterra A. (2019). Astrocyte function from information processing to cognition and cognitive impairment. Nat. Neurosci..

[13] Lobsiger C.S., Cleveland D.W. (2007). Glial cells as intrinsic components of non-cell-autonomous neurodegenerative disease. Nat. Neurosci..

[14] Hennessy E., Griffin É.W., Cunningham C. (2015). Astrocytes are primed by chronic neurodegeneration to produce exaggerated chemokine and cell infiltration responses to acute stimulation with the cytokines IL-1β and TNF-α. J. Neurosci..

[15] Tuchin V. (2016). Tissue optics and photonics: Light-tissue interaction II. J. Biomed. Photonics Eng..

[16] Anders J.J., Lanzafame R.J., Arany P.R. (2015). Low-level light/laser therapy versus photobiomodulation therapy. Photomed. Laser Surg..

[17] Zhang Z., Shen Q., Wu X., Zhang D., Xing D. (2020). Activation of PKA/SIRT1 signaling pathway by photobiomodulation therapy reduces Aβ levels in Alzheimer’s disease models. Aging Cell.

[18] Meng C., He Z., Xing D. (2013). Low-level laser therapy rescues dendrite atrophy via upregulating BDNF expression: Implications for Alzheimer’s disease. J. Neurosci..

[19] Heo J.-C., Park J.-A., Kim D.-K., Lee J.-H. (2019). Photobiomodulation (660 nm) therapy reduces oxidative stress and induces BDNF expression in the hippocampus. Sci. Rep..

[20] Song S., Zhou F., Chen W.R. (2012). Low-level laser therapy regulates microglial function through Src-mediated signaling pathways: Implications for neurodegenerative diseases. J. Neuroinflamm..

[21] Zhang D., Shen Q., Wu X., Xing D. (2021). Photobiomodulation therapy ameliorates glutamatergic dysfunction in mice with chronic unpredictable mild stress-induced depression. Oxid. Med. Cell. Longev..

[22] Ruan Y., Kato H., Taguchi Y., Yamauchi N., Umeda M. (2021). Irradiation by high-intensity red light-emitting diode enhances human bone marrow mesenchymal stem cells osteogenic differentiation and mineralization through Wnt/β-catenin signaling pathway. Lasers Med. Sci..

[23] Andreo L., Mesquita-Ferrari R.A., Grenho L., Gomes P.S., Bussadori S.K., Fernandes K.P.S., Fernandes M.H. (2021). Effects of 660-nm and 780-nm laser therapy on ST88-14 Schwann cells. Photochem. Photobiol..

[24] Jere S.W., Houreld N.N., Abrahamse H. (2018). Photobiomodulation at 660nm stimulates proliferation and migration of diabetic wounded cells via the expression of epidermal growth factor and the JAK/STAT pathway. J. Photochem. Photobiol. B.

[25] Wolfes A.C., Ahmed S., Awasthi A., Stahlberg M.A., Rajput A., Magruder D.S., Bonn S., Dean C. (2017). A novel method for culturing stellate astrocytes reveals spatially distinct Ca2+ signaling and vesicle recycling in astrocytic processes. J. Gen. Physiol..

[26] Wolfes A.C., Dean C. (2018). Culturing In vivo-like murine astrocytes using the fast, simple, and inexpensive AWESAM protocol. J. Vis. Exp..

[27] Martins D.O., Marques D.P., Venega R.A.G., Chacur M. (2020). Photobiomodulation and B vitamins administration produces antinociception in an orofacial pain model through the modulation of glial cells and cytokines expression. Brain Behav. Immun. Health.

[28] Lu Y.-Z., Fernando N., Natoli R., Madigan M., Valter K. (2018). 670 nm light treatment following retinal injury modulates Müller cell gliosis: Evidence from in vivo and in vitro stress models. Exp. Eye Res..

[29] Vinck E.M., Cagnie B.J., Cornelissen M.J., Declercq H.A., Cambier D.C. (2003). Increased fibroblast proliferation induced by light emitting diode and low power laser irradiation. Lasers Med. Sci..

[30] Wang Y., Huang Y.-Y., Wang Y., Lyu P., Hamblin M.R. (2017). Red (660 nm) or near-infrared (810 nm) photobiomodulation stimulates, while blue (415 nm), green (540 nm) light inhibits proliferation in human adipose-derived stem cells. Sci. Rep..

[31] Bergamo M.T., Vitor L.L.R., Dionísio T.J., Marques N.C.T., Oliveira R.C., Ambrosio E.C.P., Sakai V.T., Santos C.F., Lourenço Neto N., Machado M. (2021). Could the photobiomodulation therapy induce angiogenic growth factors expression from dental pulp cells?. Lasers Med. Sci..

[32] Aaron Chi-Hao C., Ying-Ying H., Praveen R.A., Michael R.H. (2009). Role of reactive oxygen species in low level light therapy. Proceedings of SPIE.

[33] Lavi R., Shainberg A., Shneyvays V., Hochauser E., Isaac A., Zinman T., Friedmann H., Lubart R. (2010). Detailed analysis of reactive oxygen species induced by visible light in various cell types. Lasers Surg. Med..

[34] de Freitas L.F., Hamblin M.R. (2016). Proposed mechanisms of photobiomodulation or low-level light therapy. IEEE J. Sel. Top. Quantum Electron..

[35] Wang Y., Huang Y.-Y., Wang Y., Lyu P., Hamblin M.R. (2016). Photobiomodulation (blue and green light) encourages osteoblastic-differentiation of human adipose-derived stem cells: Role of intracellular calcium and light-gated ion channels. Sci. Rep..

[36] Zamani A.R.N., Saberianpour S., Geranmayeh M.H., Bani F., Haghighi L., Rahbarghazi R. (2020). Modulatory effect of photobiomodulation on stem cell epigenetic memory: A highlight on differentiation capacity. Lasers Med. Sci..

[37] Van Rensburg M.J., Crous A., Abrahamse H. (2021). Potential of photobiomodulation to induce differentiation of adipose- derived mesenchymal stem cells into neural cells. Curr. Stem Cell Res. Ther..

[38] George S., Hamblin M.R., Abrahamse H. (2020). Photobiomodulation-induced differentiation of immortalized adipose stem cells to neuronal cells. Lasers Surg. Med..

[39] Mirhosseini M., Shiari R., Esmaeili Motlagh P., Farivar S. (2019). Cerebrospinal fluid and photobiomodulation effects on neural gene expression in dental pulp stem cells. J. Lasers Med. Sci..

[40] Mokoena D.R., Houreld N.N., Dhilip Kumar S.S., Abrahamse H. (2020). Photobiomodulation at 660 nm stimulates fibroblast differentiation. Lasers Surg. Med..

[41] Parpura V., Basarsky T.A., Liu F., Jeftinija K., Jeftinija S., Haydon P.G. (1994). Glutamate-mediated astrocyte-neuron signalling. Nature.

[42] Vesce S., Bezzi P., Volterra A. (1999). The active role of astrocytes in synaptic transmission. Cell. Mol. Life Sci..

[43] Durkee C.A., Araque A. (2019). Diversity and specificity of astrocyte-neuron communication. Neuroscience.

[44] Zhou B., Zuo Y.X., Jiang R.T. (2019). Astrocyte morphology: Diversity, plasticity, and role in neurological diseases. CNS Neurosci. Ther..

[45] Kim W.S., Calderhead R.G. (2011). Is light-emitting diode phototherapy (LED-LLLT) really effective?. Laser Ther..

[46] Allen S.J., Dawbarn D. (2006). Clinical relevance of the neurotrophins and their receptors. Clin. Sci..

[47] Berman M.H., Nichols T.W. (2019). Treatment of neurodegeneration: Integrating photobiomodulation and neurofeedback in Alzheimer’s dementia and Parkinson’s: A review. Photobiomodul. Photomed. Laser Surg..

[48] Hashmi J.T., Huang Y.-Y., Osmani B.Z., Sharma S.K., Naeser M.A., Hamblin M.R. (2010). Role of low-level laser therapy in neurorehabilitation. Phys. Med. Rehabil..

[49] Bouvet-Gerbettaz S., Merigo E., Rocca J.P., Carle G.F., Rochet N. (2009). Effects of low-level laser therapy on proliferation and differentiation of murine bone marrow cells into osteoblasts and osteoclasts. Lasers Surg. Med..

[50] Yang L., Tucker D., Dong Y., Wu C., Lu Y., Li Y., Zhang J., Liu T.C., Zhang Q. (2018). Photobiomodulation therapy promotes neurogenesis by improving post-stroke local microenvironment and stimulating neuroprogenitor cells. Exp. Neurol..

[51] Argibay B., Campos F., Perez-Mato M., Vieites-Prado A., Correa-Paz C., López-Arias E., da Silva-Candal A., Moreno V., Montero C., Sobrino T. (2019). Light-emitting diode photobiomodulation after cerebral ischemia. Front. Neurol..

[52] Mason M.G., Nicholls P., Cooper C.E. (2014). Re-evaluation of the near infrared spectra of mitochondrial cytochrome c oxidase: Implications for non invasive in vivo monitoring of tissues. BBA Bioenerg..

[53] Lima P.L.V., Pereira C.V., Nissanka N., Arguello T., Gavini G., Maranduba C., Diaz F., Moraes C.T. (2019). Photobiomodulation enhancement of cell proliferation at 660 nm does not require cytochrome c oxidase. J. Photochem. Photobiol. B Biol..

[54] Zhang Y., Song S., Fong C.C., Tsang C.H., Yang Z., Yang M. (2003). cDNA microarray analysis of gene expression profiles in human fibroblast cells irradiated with red light. J. Investig. Dermatol..

[55] Glass G.E. (2021). Photobiomodulation: A review of the molecular evidence for low level light therapy. J. Plastic Reconstr. Aesthet. Surg..

[56] Huang E.J., Reichardt L.F. (2001). Neurotrophins: Roles in neuronal development and function. Annu. Rev. Neurosci..

[57] Yazdani S.O., Golestaneh A.F., Shafiee A., Hafizi M., Omrani H.A., Soleimani M. (2012). Effects of low level laser therapy on proliferation and neurotrophic factor gene expression of human schwann cells in vitro. J. Photochem. Photobiol. B Biol..

[58] Yokoyama M., Black I.B., Dreyfus C.F. (1993). NGF increases brain astrocyte number in culture. Exp. Neurol..

