# Supplementary material for: Photobiomodulation with a 660-Nanometer Light-Emitting Diode Promotes Cell Proliferation in Astrocyte Culture
Source: Cells. 2021 Jul 2;10(7):1664. doi: 10.3390/cells10071664 (PMC8307591; doi:10.3390/cells10071664)
Supplement: Supplementary file 1 [file cells-10-01664-s001.zip › cells-1254032-supplementary.pdf]

cells MDPI

Supplementary Materials

Photobiomodulation with a 660-Nanometer Light-Emitting Diode Promotes Cell Proliferation in Astrocyte Culture

Sung-Ryeong Yoon [1,2,†], Namgue Hong [2,†], Min-Young Lee [3,4,*] and Jin-Chul Ahn [1,2,4,*]

1 Department of Medical Laser, Graduate School of Medicine, Dankook University, Cheonan 31116, Korea; 72201437@dankook.ac.kr
2 Medical Laser Research Center, College of Medicine, Dankook University, Cheonan 31116, Korea; hnk210@dankook.ac.kr
3 Department of Otolaryngology-Head & Neck Surgery, College of Medicine, Dankook University, Cheonan 31116, Korea
4 Beckman Laser Institute Korea, College of Medicine, Dankook University, Cheonan 31116, Korea
* Correspondence: 12180210@dankook.ac.kr (M.Y.L.); jcahn@dankook.ac.kr (J.-C.A.); Tel: +82-41-550-1785, +82-10-8884-7224 (M.Y.L.); +82-41-550-1780 (J.-C.A.); Fax: +82-41-559-7838 (M.Y.L.); +82-41-559-7838 (J.-C.A.)
† These two authors contributed equally as co-first authors.

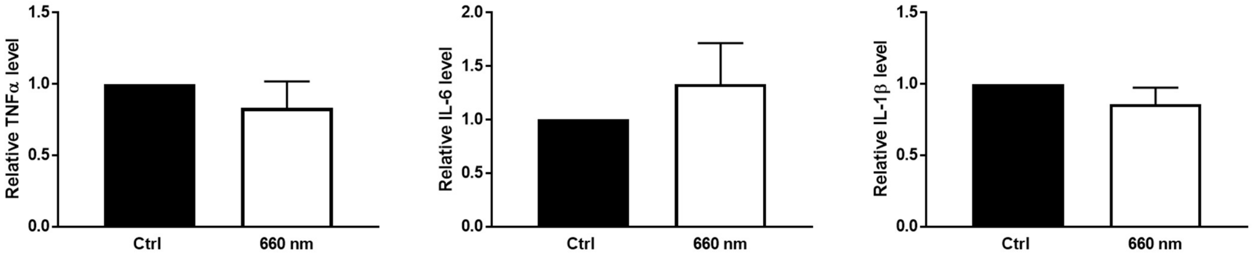

Figure S1. Western blot analysis of pro-inflammatory cytokines (*n* = 4). All full-length blots/gels are presented in Supplementary Figure S2.

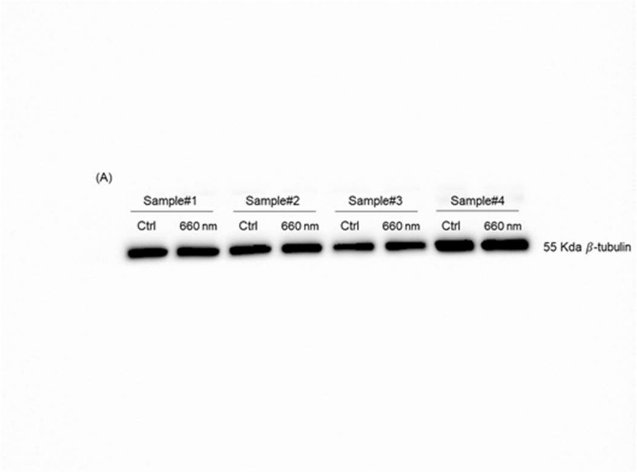
(A)
Sample#1
Sample#2
Sample#3
Sample#4
Ctrl
660 nm
55 Kda β-tubulin

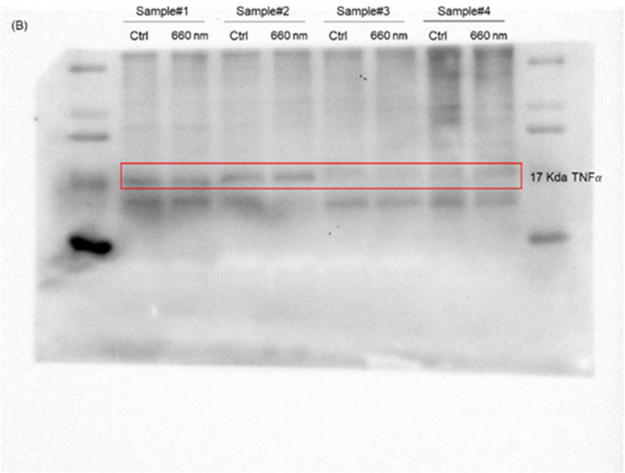
(B)
Sample#1
Sample#2
Sample#3
Sample#4
Ctrl
660 nm
17 Kda TNFα

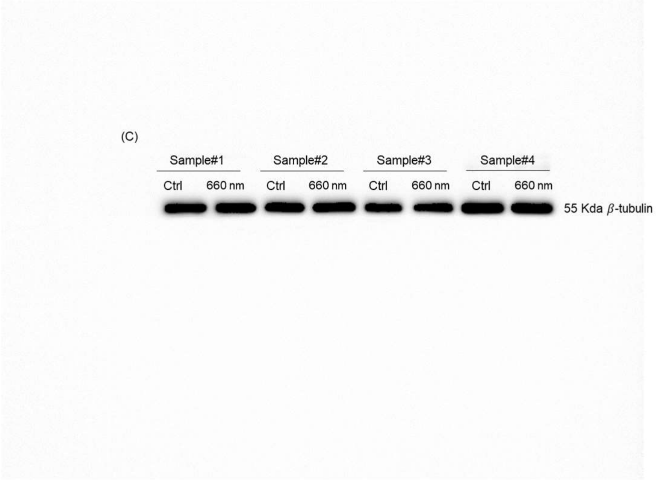
(C)
Sample#1
Sample#2
Sample#3
Sample#4
Ctrl
660 nm
55 Kda β-tubulin

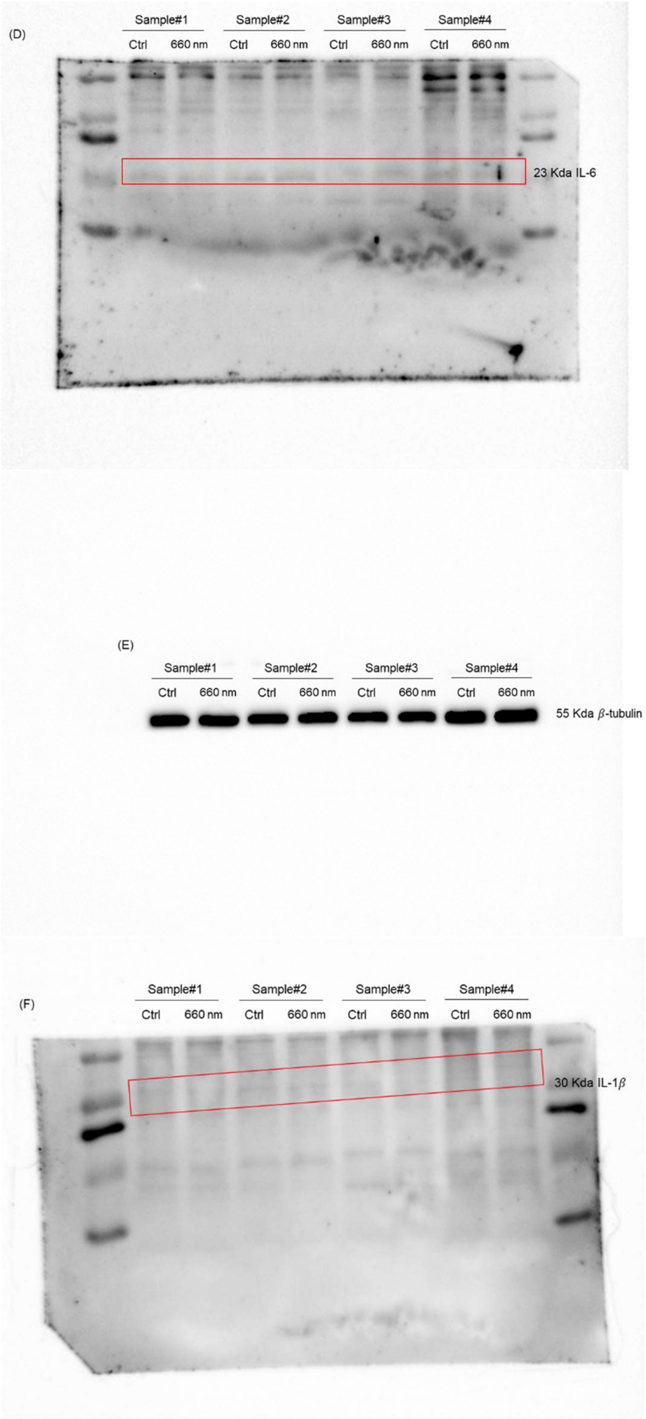

Figure S2. Full-length blots of pro-inflammatory cytokines. (**A**) β-tubulin of TNFα, (**B**) TNFα, (**C**) β-tubulin of IL-6, (**D**) IL-6, (**E**) IL-1β, (**F**) β-tubulin of IL-1β.

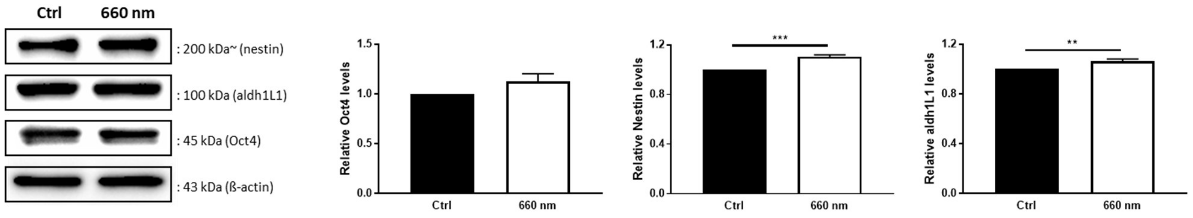

Figure S3. Aldh1L1 of gels/blots was Western blot analysis of proteins related to differentiation of astrocyte ($n = 4$; ** $p < 0.01$; *** $p < 0.001$). Cropped from different gels. All full-length blots/gels are presented in Supplementary Figure S4.

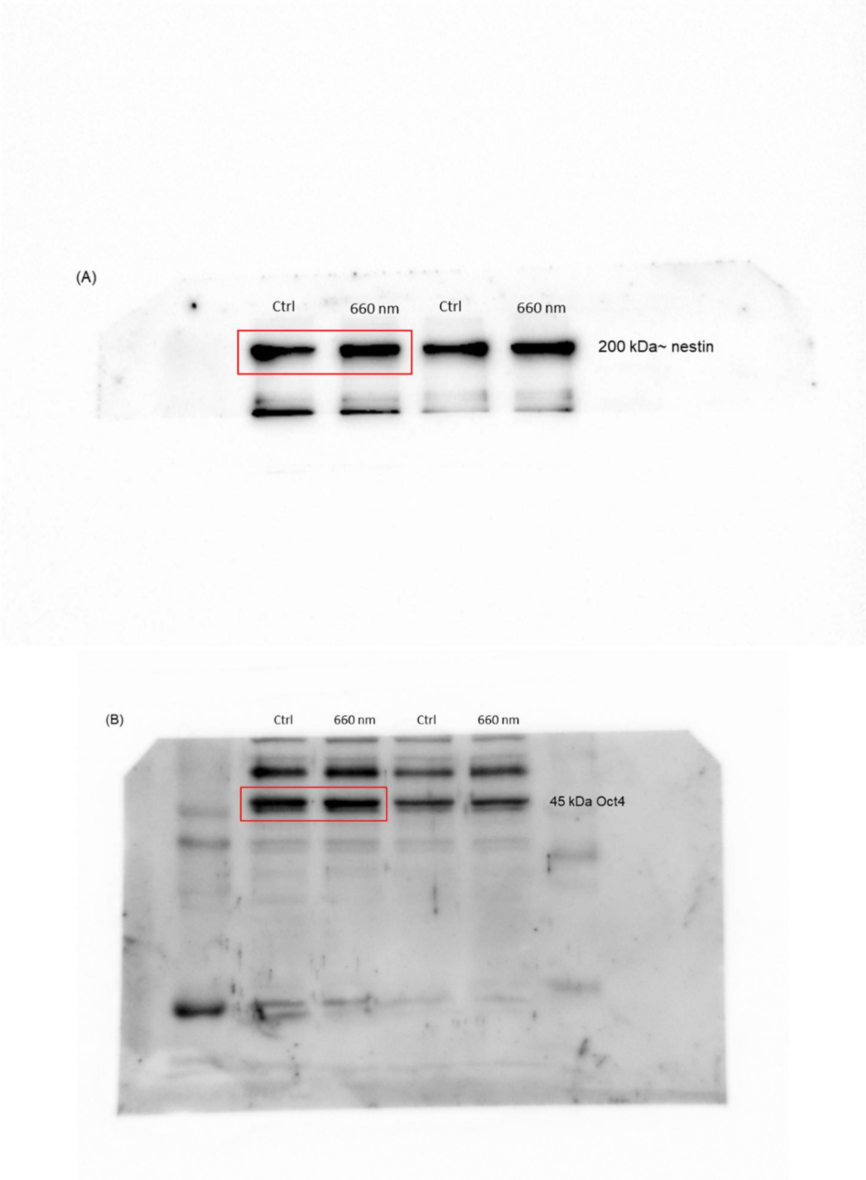

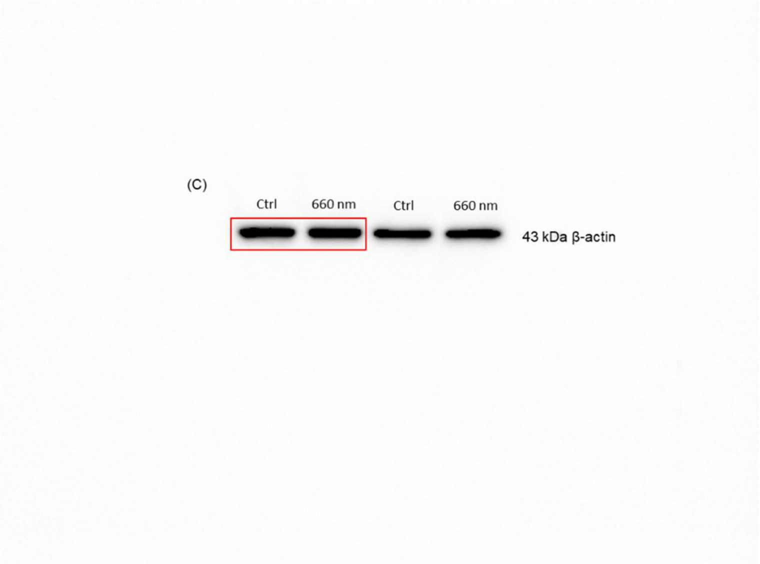

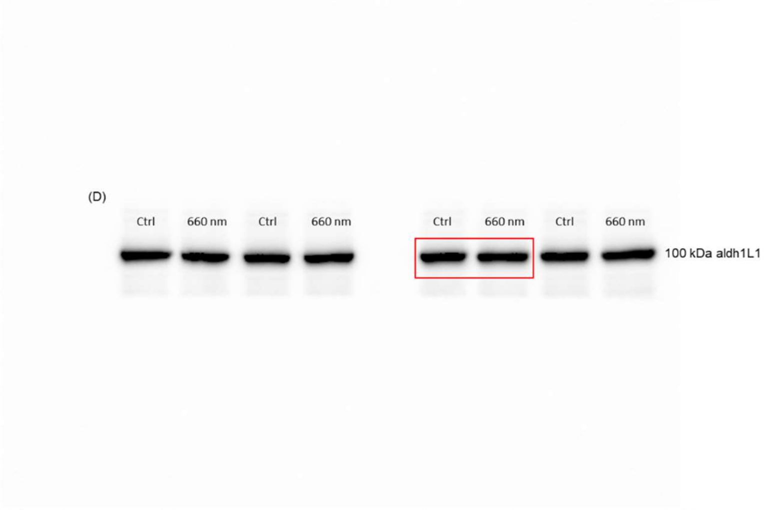

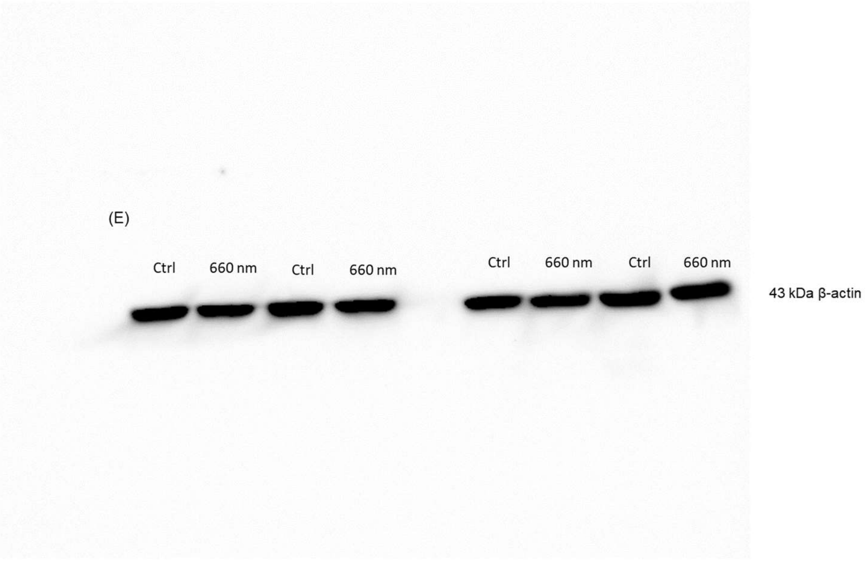

Figure S4. Full-length blots of differentiation of astrocyte Western blot analysis. (**A**) nestin, (**B**) Oct4, (**C**) β-actin, (**D**) aldh1L1, (**E**) β-actin.

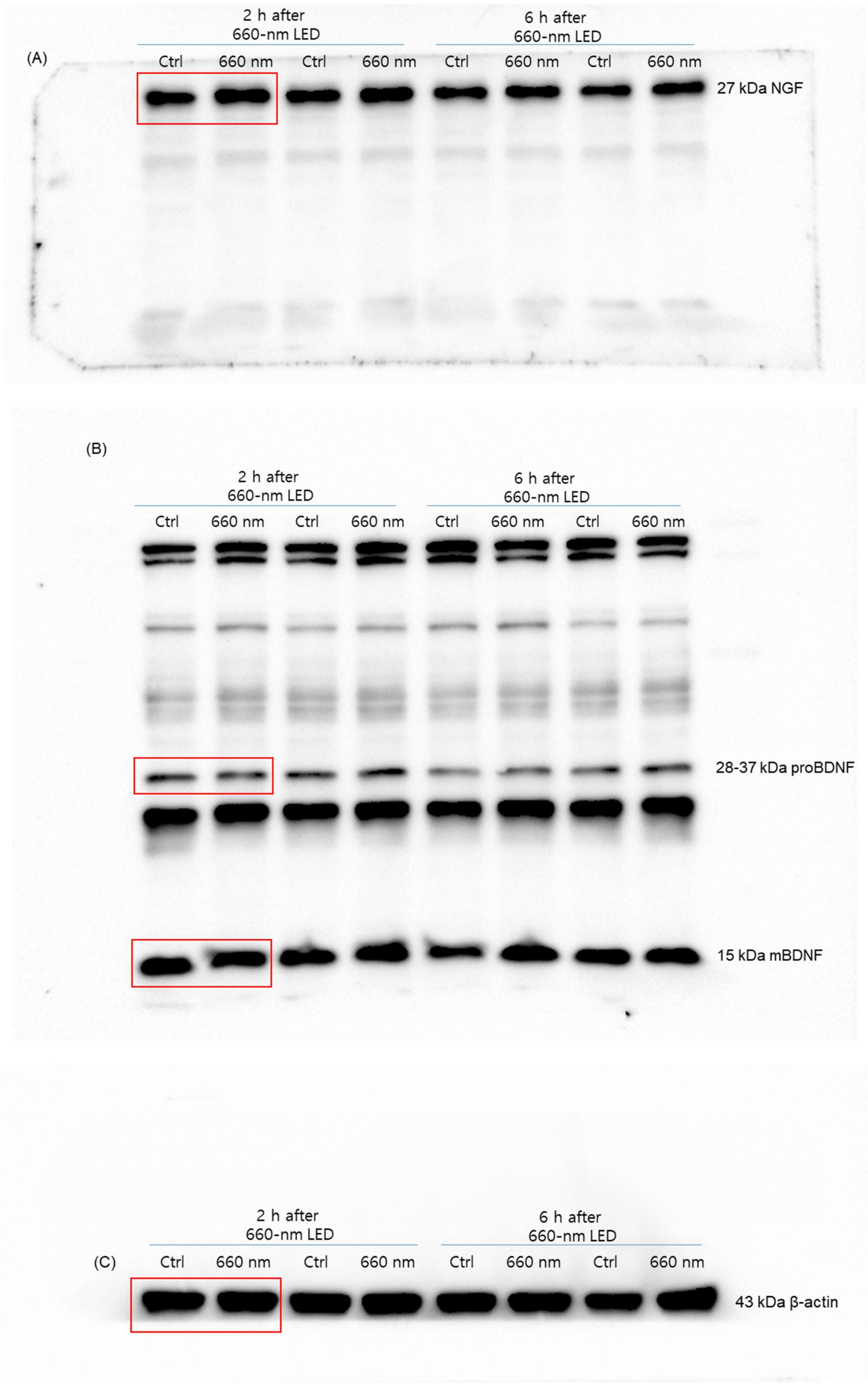

Figure S5. Full-length blots of neurotrophic factor proteins. (**A**) NGF, (**B**) proBDNF and mBDNF, (**C**) β-actin.
